# Composition Profiler: a tool for discovery and visualization of amino acid composition differences

**DOI:** 10.1186/1471-2105-8-211

**Published:** 2007-06-19

**Authors:** Vladimir Vacic, Vladimir N Uversky, A Keith Dunker, Stefano Lonardi

**Affiliations:** 1 Department of Computer Science and Engineering, University of California, Riverside, CA 92521, USA; 2 Center for Computational Biology and Bioinformatics, Department of Biochemistry and Molecular Biology, Indiana University School of Medicine, Indianapolis, IN 46202, USA; 3 Institute for Biological Instrumentation, Russian Academy of Sciences, 142290 Pushchino, Moscow Region, Russia

## Abstract

**Background:**

Composition Profiler is a web-based tool for semi-automatic discovery of enrichment or depletion of amino acids, either individually or grouped by their physico-chemical or structural properties.

**Results:**

The program takes two samples of amino acids as input: a query sample and a reference sample. The latter provides a suitable background amino acid distribution, and should be chosen according to the nature of the query sample, for example, a standard protein database (e.g. SwissProt, PDB), a representative sample of proteins from the organism under study, or a group of proteins with a contrasting functional annotation. The results of the analysis of amino acid composition differences are summarized in textual and graphical form.

**Conclusion:**

As an exploratory data mining tool, our software can be used to guide feature selection for protein function or structure predictors. For classes of proteins with significant differences in frequencies of amino acids having particular physico-chemical (e.g. hydrophobicity or charge) or structural (e.g. α helix propensity) properties, Composition Profiler can be used as a rough, light-weight visual classifier.

## Background

Often the first step in characterizing a group of related non-homologous proteins (that is, for which there is no meaningful multiple sequence alignment) is to identify statistically significant patterns of amino acid enrichment or depletion. Here we introduce Composition Profiler, a web-based software that automates this task and graphically summarizes the results. Composition Profiler is also available as a stand-alone command line application that can be used for task automation or analysis of large samples. The following sections will introduce the methodology and discuss several examples of composition profiles in greater depth.

## Methods

### Fractional differences

Let *P *denote the protein sample under study, *Q *the background sample, and let *p*_*k *_and *q*_*k *_denote the probabilities of observing amino acid *k *in the two samples. Let us assume that the amino acid compositions of the two samples *P *and *Q *are independent and identically distributed, each generated by a separate stochastic process according to probability distributions *p *= (*p*_*Ala*_, *p*_*Arg*_, ...) and *q *= (*q*_*Ala*_, *q*_*Arg*_, ...). The probability distributions *p *and *q *are estimated by computing the means and confidence intervals of the relative frequencies of residues observed over a set of pseudo-replicate datasets obtained by bootstrap sampling of whole proteins from the original samples *P *and *Q*. We define the fractional difference *h *between distributions *p *and *q *as

hk=pk−qkqk,where k=Ala,Arg, ...
 MathType@MTEF@5@5@+=feaafiart1ev1aaatCvAUfKttLearuWrP9MDH5MBPbIqV92AaeXatLxBI9gBaebbnrfifHhDYfgasaacH8akY=wiFfYdH8Gipec8Eeeu0xXdbba9frFj0=OqFfea0dXdd9vqai=hGuQ8kuc9pgc9s8qqaq=dirpe0xb9q8qiLsFr0=vr0=vr0dc8meaabaqaciaacaGaaeqabaqabeGadaaakeaafaqabeqacaaabaGaemiAaG2aaSbaaSqaaiabdUgaRbqabaGccqGH9aqpdaWcaaqaaiabdchaWnaaBaaaleaacqWGRbWAaeqaaOGaeyOeI0IaemyCae3aaSbaaSqaaiabdUgaRbqabaaakeaacqWGXbqCdaWgaaWcbaGaem4AaSgabeaaaaGccqGGSaalaeaacqqG3bWDcqqGObaAcqqGLbqzcqqGYbGCcqqGLbqzcqqGGaaicqWGRbWAcqGH9aqpcqqGbbqqcqqGSbaBcqqGHbqycqGGSaalcqqGbbqqcqqGYbGCcqqGNbWzcqGGSaalcqqGGaaicqGGUaGlcqGGUaGlcqGGUaGlaaaaaa@523A@

**Figure 1:**
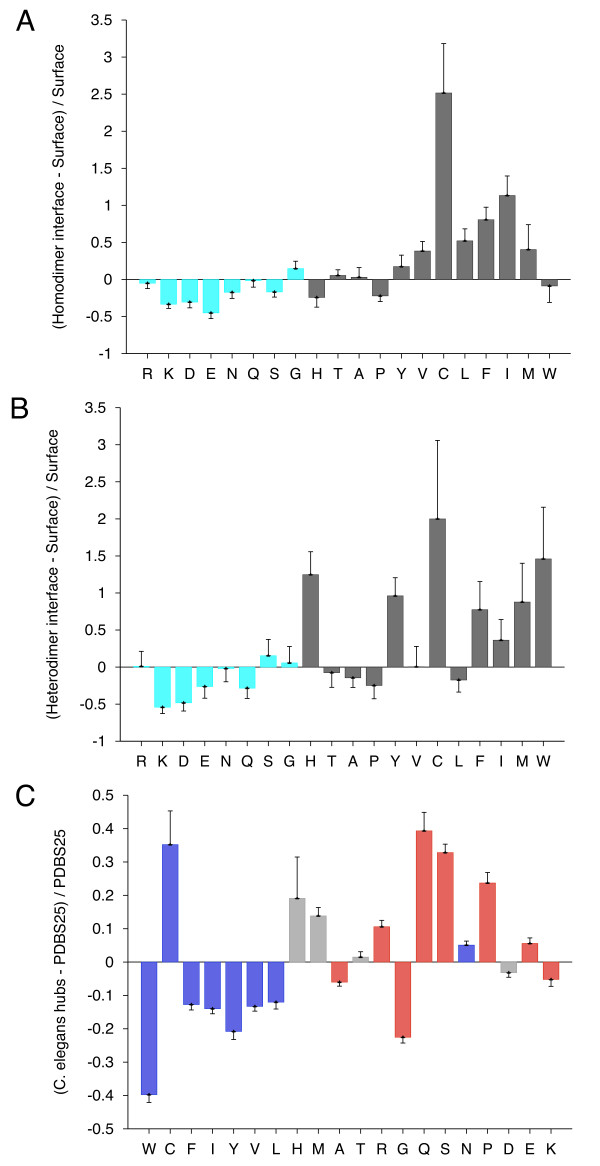
**Composition Profiles of homo- (A) and heterodimerisation (B) interfaces and hub proteins from *C. elegans *PPI network**. Analysis of residues in homo- and heterodimer interfaces against surface residues of monomeric proteins shows slight depletion in hydrophilics (cyan) and enrichement in hydrophobics (black) as a general trend, although homodimer interfaces show closer resemblance to the protein surfaces. Composition profile of hub proteins shows a general enrichment in disorder (red) and depletion in order promoting residues (blue).

**Figure 2:**
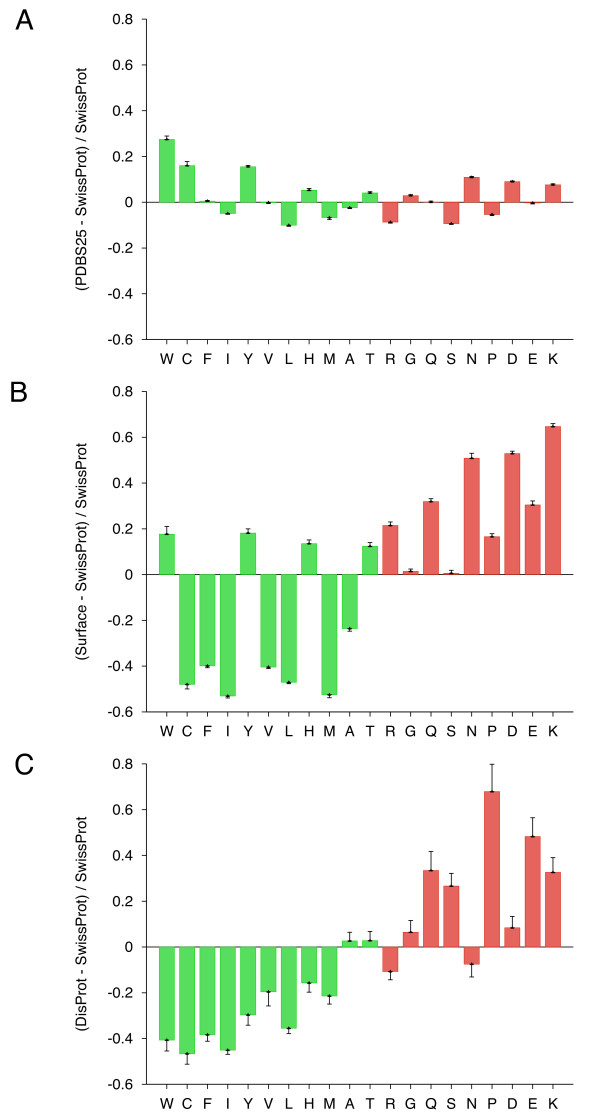
**Composition profiles of PDB Select 25 (A), surface residues of monomers (B) and DisProt (C) against SwissProt**. Plotting the three graphs using the same y-axis scale, same ordering of amino acids and the same color-coding scheme (flexibility) allows for a direct visual comparison between enrichment and depletion patterns in the three datasets.

Figures 1 and 2 show several examples of compositional difference plots produced by Composition Profiler. The values for *h*_*k *_are displayed as bar heights, and the error bars *e*_*k *_represent fractional differences of the standard deviations of observed relative frequencies of the bootstrap samples. More precisely

ek=(pk+σp,k)−(qk+σq,k)qk+σq,k−hk
 MathType@MTEF@5@5@+=feaafiart1ev1aaatCvAUfKttLearuWrP9MDH5MBPbIqV92AaeXatLxBI9gBaebbnrfifHhDYfgasaacH8akY=wiFfYdH8Gipec8Eeeu0xXdbba9frFj0=OqFfea0dXdd9vqai=hGuQ8kuc9pgc9s8qqaq=dirpe0xb9q8qiLsFr0=vr0=vr0dc8meaabaqaciaacaGaaeqabaqabeGadaaakeaacqWGLbqzdaWgaaWcbaGaem4AaSgabeaakiabg2da9maalaaabaGaeiikaGIaemiCaa3aaSbaaSqaaiabdUgaRbqabaGccqGHRaWkiiGacqWFdpWCdaWgaaWcbaGaemiCaaNaeiilaWIaem4AaSgabeaakiabcMcaPiabgkHiTiabcIcaOiabdghaXnaaBaaaleaacqWGRbWAaeqaaOGaey4kaSIae83Wdm3aaSbaaSqaaiabdghaXjabcYcaSiabdUgaRbqabaGccqGGPaqkaeaacqWGXbqCdaWgaaWcbaGaem4AaSgabeaakiabgUcaRiab=n8aZnaaBaaaleaacqWGXbqCcqGGSaalcqWGRbWAaeqaaaaakiabgkHiTiabdIgaOnaaBaaaleaacqWGRbWAaeqaaaaa@5554@

where *σ *_*p*,*k *_and *σ *_*q*,*k *_are standard deviations of frequencies of amino acid *k *in bootstrap samples based on *P *and *Q*, respectively.

Statistical significance associated with a specific value of *h*_*k *_is estimated using the two-sample t-test between two sequences of binary indicator variables, one sequence for each of the samples *P *and *Q*. A particular *h*_*k *_is statistically significant when the lowest value at which the null hypothesis that the same underlying Gaussian distribution generated both *P *and *Q *can be rejected, is smaller than a user-specified statistical significance (α) value. To avoid spurious significance which may appear by chance alone due to the number of statistical tests performed, the conservative Bonferroni correction can be optionally used to adjust the test-wise significance cut-off by dividing the α-value by the number of individual significance tests performed.

### Relative entropy

Fractional differences provide a detailed, per amino acid, characterization of the dissimilarity between two samples. However, there are situations when it is useful to summarize the degree of dissimilarity into a single value, for example, when a large number of samples need to be compared against each other to determine pairwise similarities. Relative entropy (also known as Kullback-Leibler divergence, information divergence, or information gain) is an information theoretical measure that quantifies the distance between two probability distributions. Using the frequencies of residues in samples *P *and *Q *as the maximum likelihood estimate for the underlying probability distributions *p *and *q*, the *relative entropy *of the sample *P *with respect to the sample *Q *is defined as

H(p||q)=∑kpklog⁡(pkqk),where k=Ala,Arg, ...
 MathType@MTEF@5@5@+=feaafiart1ev1aaatCvAUfKttLearuWrP9MDH5MBPbIqV92AaeXatLxBI9gBaebbnrfifHhDYfgasaacH8akY=wiFfYdH8Gipec8Eeeu0xXdbba9frFj0=OqFfea0dXdd9vqai=hGuQ8kuc9pgc9s8qqaq=dirpe0xb9q8qiLsFr0=vr0=vr0dc8meaabaqaciaacaGaaeqabaqabeGadaaakeaafaqabeqacaaabaGaemisaGKaeiikaGIaemiCaaNaeiiFaWNaeiiFaWNaemyCaeNaeiykaKIaeyypa0ZaaabeaeaacqWGWbaCdaWgaaWcbaGaem4AaSgabeaakiGbcYgaSjabc+gaVjabcEgaNnaabmaabaWaaSaaaeaacqWGWbaCdaWgaaWcbaGaem4AaSgabeaaaOqaaiabdghaXnaaBaaaleaacqWGRbWAaeqaaaaaaOGaayjkaiaawMcaaaWcbaGaem4AaSgabeqdcqGHris5aOGaeiilaWcabaGaee4DaCNaeeiAaGMaeeyzauMaeeOCaiNaeeyzauMaeeiiaaIaem4AaSMaeyypa0JaeeyqaeKaeeiBaWMaeeyyaeMaeiilaWIaeeyqaeKaeeOCaiNaee4zaCMaeiilaWIaeeiiaaIaeiOla4IaeiOla4IaeiOla4caaaaa@5FEF@

Relative entropy is always non-negative, and its value reaches zero only when two amino acid distributions are identical. It is not symmetric, that is, *H *(*p *|| *q*) is not necessarily equal to *H *(*q *|| *p*).

Statistical significance of the observed relative entropy value between *P *and *Q *was evaluated using relative entropy as the test statistic. Under the null hypothesis that amino acid compositions of the two samples came from the same underlying distribution, the p-value is estimated as

p−value=∑i=1nI(H(p^i||q^i)≥H(p||q))n
 MathType@MTEF@5@5@+=feaafiart1ev1aaatCvAUfKttLearuWrP9MDH5MBPbIqV92AaeXatLxBI9gBaebbnrfifHhDYfgasaacH8akY=wiFfYdH8Gipec8Eeeu0xXdbba9frFj0=OqFfea0dXdd9vqai=hGuQ8kuc9pgc9s8qqaq=dirpe0xb9q8qiLsFr0=vr0=vr0dc8meaabaqaciaacaGaaeqabaqabeGadaaakeaacqqGWbaCcqGHsislcqqG2bGDcqqGHbqycqqGSbaBcqqG1bqDcqqGLbqzcqGH9aqpdaWcaaqaamaaqadabaGaemysaK0aaeWaaeaacqWGibascqGGOaakcuWGWbaCgaqcamaaBaaaleaacqWGPbqAaeqaaOGaeiiFaWNaeiiFaWNafmyCaeNbaKaadaWgaaWcbaGaemyAaKgabeaakiabcMcaPiabgwMiZkabdIeaijabcIcaOiabdchaWjabcYha8jabcYha8jabdghaXjabcMcaPaGaayjkaiaawMcaaaWcbaGaemyAaKMaeyypa0JaeGymaedabaGaemOBa4ganiabggHiLdaakeaacqWGUbGBaaaaaa@5804@

where p^i
 MathType@MTEF@5@5@+=feaafiart1ev1aaatCvAUfKttLearuWrP9MDH5MBPbIqV92AaeXatLxBI9gBaebbnrfifHhDYfgasaacH8akY=wiFfYdH8Gipec8Eeeu0xXdbba9frFj0=OqFfea0dXdd9vqai=hGuQ8kuc9pgc9s8qqaq=dirpe0xb9q8qiLsFr0=vr0=vr0dc8meaabaqaciaacaGaaeqabaqabeGadaaakeaacuWGWbaCgaqcamaaBaaaleaacqWGPbqAaeqaaaaa@2FAC@ and q^i
 MathType@MTEF@5@5@+=feaafiart1ev1aaatCvAUfKttLearuWrP9MDH5MBPbIqV92AaeXatLxBI9gBaebbnrfifHhDYfgasaacH8akY=wiFfYdH8Gipec8Eeeu0xXdbba9frFj0=OqFfea0dXdd9vqai=hGuQ8kuc9pgc9s8qqaq=dirpe0xb9q8qiLsFr0=vr0=vr0dc8meaabaqaciaacaGaaeqabaqabeGadaaakeaacuWGXbqCgaqcamaaBaaaleaacqWGPbqAaeqaaaaa@2FAE@ are amino acid compositions of pseudo-replicate datasets obtained by bootstrap sampling of whole proteins from the original samples *P *and *Q*, *I(t) *is the indicator variable which takes the value 1 if the condition *t *is true, and 0 otherwise, and *n *is the total number of bootstrap iterations.

### Background distributions

Composition Profiler provides composition statistics for four standard amino acid datasets, computed as means and standard deviations over 100,000 bootstrap iterations, to be used as background distributions (see Table 1). These datasets are: (1) SwissProt 51 [1], most similar to the distribution of amino acids in nature out of the four; (2) PDB Select 25, a subset of structures from the Protein Data Bank [2] with less than 25% sequence identity, biased towards the composition of proteins amenable to crystallization studies; (3) surface residues determined by the Molecular Surface Package [3] over a sample of PDB structures of monomeric proteins, suitable for analyzing phenomena on protein surfaces, such as binding; and (4) DisProt 3.4, is a set of consensus sequences of experimentally determined disordered regions [4].

**Table 1:** Residue compositions of four protein datasets. The values are means and standard deviations of relative frequencies obtained in 100,000 bootstrap sampling iterations

Residue\%	SwissProt	PDB S25	Surface Residues	DisProt
Ala (A)	7.89 ± 0.05	7.70 ± 0.08	6.03 ± 0.13	8.10 ± 0.35
Arg (R)	5.40 ± 0.04	4.93 ± 0.06	6.56 ± 0.13	4.82 ± 0.23
Asn (N)	4.13 ± 0.04	4.58 ± 0.06	6.23 ± 0.15	3.82 ± 0.27
Asp (D)	5.35 ± 0.03	5.83 ± 0.05	8.18 ± 0.10	5.80 ± 0.30
Cys (C)	1.50 ± 0.02	1.74 ± 0.05	0.78 ± 0.04	0.80 ± 0.08
Gln (Q)	3.95 ± 0.03	3.95 ± 0.05	5.21 ± 0.09	5.27 ± 0.37
Glu (E)	6.67 ± 0.04	6.65 ± 0.07	8.70 ± 0.17	9.89 ± 0.61
Gly (G)	6.96 ± 0.04	7.16 ± 0.07	7.06 ± 0.11	7.41 ± 0.40
His (H)	2.29 ± 0.02	2.41 ± 0.04	2.60 ± 0.06	1.93 ± 0.11
Ile (I)	5.90 ± 0.04	5.61 ± 0.06	2.77 ± 0.07	3.24 ± 0.13
Leu (L)	9.65 ± 0.04	8.68 ± 0.08	5.11 ± 0.08	6.22 ± 0.25
Lys (K)	5.92 ± 0.05	6.37 ± 0.08	9.75 ± 0.16	7.85 ± 0.45
Met (M)	2.38 ± 0.02	2.22 ± 0.04	1.13 ± 0.04	1.87 ± 0.10
Phe (F)	3.96 ± 0.03	3.98 ± 0.04	2.38 ± 0.05	2.44 ± 0.13
Pro (P)	4.83 ± 0.03	4.57 ± 0.05	5.63 ± 0.10	8.11 ± 0.63
Ser (S)	6.83 ± 0.04	6.19 ± 0.06	6.87 ± 0.13	8.65 ± 0.43
Thr (T)	5.41 ± 0.02	5.63 ± 0.05	6.08 ± 0.11	5.56 ± 0.24
Trp (W)	1.13 ± 0.01	1.44 ± 0.03	1.33 ± 0.05	0.67 ± 0.06
Tyr (Y)	3.03 ± 0.02	3.50 ± 0.04	3.58 ± 0.08	2.13 ± 0.15
Val (V)	6.73 ± 0.03	6.72 ± 0.06	4.01 ± 0.06	5.41 ± 0.44

Depending on the nature of the query sample, other suitable background distributions might be representative samples of proteins from the organisms under study, or samples of proteins with contrasting functional annotation.

### Physico-chemical and structural properties

In addition to the ability to determine enrichment or depletion patterns of individual amino acids, Composition Profiler can also detect enrichment or depletion of groups of amino acids classified by aromaticity, charge, polarity (Zimmerman index [5]), hydrophobicity (indices of Eisenberg [6], Kyte and Doolittle [7], and Fauchere and Pliska [8]), flexibility (Vihinen scale [9]), surface exposure (Janin scale [10]), solvation potential [11], interface propensity [12], normalized frequency of occurrence in α helices, β structures, and coils [13], linker [14] and disorder [15] propensities, size [16] and bulkiness [5].

## Results

The graphical output of Composition Profiler is a bar chart composed of twenty data points, one for each amino acid (see Figure 1), where bar heights indicate enrichment or depletion and error bars correspond to one standard deviation, as described in equations 1 and 2. The output is designed to assist the discovery of statistically significant composition anomalies by color-coding and sorting residues according to their physico-chemical or structural properties. For example, if the property being tested is flexibility, the tool will group rigid amino acids on the left hand side of the plot and flexible amino acids on the right hand side of the plot.

When run in discovery mode, Composition Profiler will test all groupings of amino acids according to the listed properties for statistically significant differences between the two samples. The discovery mode uses a two-sample t-test between two sequences of binary indicator variables (e.g. for flexibility, indicator variable would be 1 if the residue is flexible, and 0 if it is rigid).

In the following sections we examine composition profiles of several groups of proteins and discuss general trends observed.

### Heterodimer interfaces

Protein-protein interaction sites have been intensively studied in an attempt to understand the molecular determinants of protein recognition and to identify specific characteristics of the interactions, such as residue propensities, residue pairing preferences, hydrophobicity, size, shape, solvent accessibility, and hydrogen bond protection. Homocomplexes, for example, are often permanent and optimized, whereas many heterocomplexes are nonobligatory, associating and disassociating according to the environmental or external factors and involve proteins that must also exist independently [11]. Figures 1A and 1B give composition profiles of interface residues of homodimers and heterodimers in comparison to the amino acid composition of surfaces of monomeric proteins. Both kinds of interfaces are generally enriched in hydrophobic residues (right hand side of the graph), which in part explains their propensity towards complexation. Interfaces of heterodimers are enriched in polar histidine and tyrosine, which is consistent with the finding that transient protein-protein complex interfaces are more polar than those of stable oligomeric proteins [11,12,17]. Heterocomplex interfaces are enriched in all three major aromatics (trypthopan, tyrosine, and phenylalanine), as these three residues are bulky, planar and rigid which enhances the prospects for binding.

### Hub proteins of *C. elegans *PPI network

A potential association between protein connectivity and protein intrinsic disorder was studied for proteins with various numbers of interacting partners from four eukaryotic organisms (*C. elegans*, *S. cerevisiae*, *D. melanogaster*, and *H. sapiens*) [18]. A more detailed analysis revealed that hub proteins, defined as proteins interacting with at least 10 partners, are significantly more disordered than end proteins, defined as those that interact with just one partner. To test the compositional bias of hubs and ends, the fractional difference between hubs and ends compositions and PDBS25 compositions was calculated. This analysis revealed that that hubs are enriched in many of the disorder-promoting amino acids, whereas compositions of ends were shown to be relatively close to that of ordered proteins. This study demonstrated that intrinsic disorder is a distinctive and common characteristic of eukaryotic hub proteins, and that disorder may serve as a determinant of protein interactivity. This particular example (Figure 1C) shows the composition profile of hub proteins from *C. elegans*. The red-colored bars on the right hand side of the graph represent disorder-promoting residues.

## Discussion

The need for analyzing sequences against an appropriate background can best be illustrated by running Composition Profiler on any of the four standard distributions against the remaining three and observing the differences in composition. Surface residues from monomeric proteins (Figure 2B) and regions of protein disorder (Figure 2C) generally show depletion in low flexibility (according to the Vihinen scale [9]) and enrichment in high flexibility residues. Unlike the disordered region dataset, surface residues are enriched in tryptophan, tyrosine (both order-promoting) and histidine (disorder-neutral). One of possible explanations for this is the preference for their presence in the active sites, where those bulky and planar residues may provide geometric restrictions and help in establishing appropriate contacts with substrates or ligands. In comparison with Swiss-Prot, proteins from PDB Select 25 (Figure 2A) are enriched in the major order-promoting residues (tryptophan, cysteine, and tyrosine) and depleted in disorder-promoting residues (arginine, serine, and proline). It is of interest to observe the enrichment of disorder-promoting residues such as asparagine, aspartic acid, and lysine in PDB Select 25 proteins.

## Conclusion

The notion of fractional difference as a measurement of the relative variation between the two samples was first employed by Romero *et al*. [19]. It has since been used in studies of cell-signalling and cancer-associated proteins [20], serine/arginine-rich splicing factors [21] and hub proteins of PPI networks [18], among others.

As an exploratory data mining tool, our software can be used to guide feature selection for protein function or structure predictors – good features are ones that discriminate well between the two samples. For classes of proteins which show enrichment in amino acids having particular physico-chemical properties, Composition Profiler can be thought of as a rough, light-weight visual classifier. For example, composition profiles with fractional differences which show enrichment in disorder-promoting residues constitute strong indications of intrinsic disorder [15].

## Availability and requirements

**Project name: **Composition Profiler

**Project home page: **

**Operating system(s): **Linux, Mac OS X

**Programming language: **Ruby, C, C++

**Other requirements: **GhostScript, ImageMagick, gnuplot 4.2, Apache web server

**License: **MIT Open Source License

**Any restrictions to use by non-academics: **none

## Authors' contributions

AKD originated the fractional difference method. VV wrote the application and drafted the manuscript. VNU provided relevant biological examples. SL, VNU, and AKD helped in drafting the manuscript. All authors read and approved the final manuscript.

## References

[B1] Bairoch A, Apweiler R, Wu CH, Barker WC, Boeckmann B, Ferro S, Gasteiger E, Huang H, Lopez R, Magrane M, Martin MJ, Natale DA, O'Donovan C, Redaschi N, Yeh LS (2005). The Universal Protein Resource (UniProt). Nucleic Acids Research.

[B2] Berman HM, Westbrook J, Feng Z, Gilliland G, Bhat TN, Weissig H, Shindyalov IN, Bourne PE (2000). The Protein Data Bank. Nucleic Acids Research.

[B3] Molecular Surface Package. http://www.biohedron.com.

[B4] Sickmeier M, Hamilton JA, LeGall T, Vacic V, Cortese MS, Tantos A, Szabo B, Tompa P, Chen J, Uversky VN, Obradovic Z, Dunker AK (2007). DisProt: the Database of Disordered Proteins. Nucleic Acids Research.

[B5] Zimmerman JM, Eliezer N, Simha R (1968). The characterization of amino acid sequences in proteins by statistical methods. J Theor Biol.

[B6] Eisenberg D, Schwarz E, Komaromy M, Wall R (1984). Analysis of membrane and surface protein sequences with the hydrophobic moment plot. J Molecular Biology.

[B7] Kyte J, Doolittle RF (1982). simple method for displaying the hydropathic character of a protein. J Molecular Biology.

[B8] Fauchere J-L, Pliska VE (1983). Hydrophobic parameters pi of amino acid side chains from partitioning of N-acetyl-amino-acid amides. Eur J Med Chem.

[B9] Vihinen M, Torkkila E, Riikonen P (1994). Accuracy of protein flexibility predictions. Proteins.

[B10] Janin J (1979). Surface and inside volumes in globular proteins. Nature.

[B11] Jones S, Thornton J (1997). Analysis of protein-proteins interaction sites using surface patches. J Molecular Biology.

[B12] Jones S, Thornton J (1996). Principles of protein-protein interactions. Proc Natl Acad Sci USA.

[B13] Nagano K (1973). Local analysis of the mechanism of protein folding. I. Prediction of helices, loops, and beta-structures from primary structure. J Mol Biol.

[B14] George RA, Heringa J (2003). An analysis of protein domain linkers: their classification and role in protein folding. Protein Eng.

[B15] Dunker AK, Lawson JD, Brown CJ, Williams RM, Romero P, Oh JS, Oldfield CJ, Campen AM, Ratliff CM, Hipps KW, Ausio J, Nissen MS, Reeves R, Kang C, Kissinger CR, Bailey RW, Griswold MD, Chiu W, Garner EC, Obradovic Z (2001). Intrinsically disordered protein. J Mol Graph Model.

[B16] Dawson DM, Brock DJH, Mayo O (1972). The Biochemical Genetics of Man.

[B17] Valdar WS, Thornton JM (2001). Protein-protein interfaces: analysis of amino acid conservation in homodimers. Proteins.

[B18] Haynes C, Oldfield CJ, Ji F, Klitgord N, Cusick ME, Radivojac P, Uversky VN, Vidal M, Iakoucheva LM (2006). Intrinsic disorder is a common feature of hub proteins from four eukaryotic interactomes. PLoS Computational Biology.

[B19] Romero P, Obradovic Z, Li X, Garner EC, Brown CJ, Dunker AK (2001). Sequence complexity of disordered protein. Proteins.

[B20] Iakoucheva LM, Brown CJ, Lawson JD, Obradovic Z, Dunker AK (2002). Intrinsic disorder in cell-signaling and cancer-associated proteins. J Molecular Biology.

[B21] Haynes C, Iakoucheva LM (2006). Serine/arginine-rich splicing factors belong to a class of intrinsically disordered proteins. Nucleic Acids Research.

[B22] Cephes Math Library. http://www.netlib.org/cephes.

